# Orodispersible Films—Current State of the Art, Limitations, Advances and Future Perspectives

**DOI:** 10.3390/pharmaceutics15020361

**Published:** 2023-01-20

**Authors:** Jan Ferlak, Weronika Guzenda, Tomasz Osmałek

**Affiliations:** 1 Chair and Department of Pharmaceutical Technology, Poznan University of Medical Sciences, 6 Grunwaldzka Street, 60-780 Poznan, Poland; 2 Pharmacy Practice Division, Chair and Department of Pharmaceutical Technology, Poznan University of Medical Sciences, 6 Grunwaldzka Street, 60-780 Poznan, Poland

**Keywords:** orodispersible films, drug delivery, pharmaceutical manufacturing, polymers

## Abstract

Orodispersible Films (ODFs) are drug delivery systems manufactured with a wide range of methods on a big scale or for customized medicines and small-scale pharmacy. Both ODFs and their fabrication methods have certain limitations. Many pharmaceutical companies and academic research centers across the world cooperate in order to cope with these issues and also to find new formulations for a wide array of APIs what could make their work profitable for them and beneficial for patients as well. The number of pending patent applications and granted patents with their innovative approaches makes the progress in the manufacturing of ODFs unquestionable. The number of commercially available ODFs is still growing. However, some of them were discontinued and are no longer available on the markets. This review aims to summarize currently marketed ODFs and those withdrawn from sale and also provides an insight into recently published studies concerning orodispersible films, emphasizing of utilized APIs. The work also highlights the attempts of scientific communities to overcome ODF’s manufacturing methods limitations.

## 1. Introduction

Tablets and capsules are commonly used solid oral drug formulations. For some patients, however, using these dosage forms on a daily basis is quite problematic and sometimes even impossible. There are some groups of people with swallowing difficulties, a fear of choking or dysphagia. These problems might be experienced by geriatric, pediatric, or mentally ill patients, people after anesthesia, with Parkinson’s disease, or Alzheimer disease [1,2,3,4,5]. There are also other reasons for avoiding taking tablets or capsules such as nausea or a reduced water-intake plan for some patients. Additionally, limited access to the water that is sometimes experienced by travelers might be problematic, as tablets or capsules need water for the proper disintegration and releasing of active pharmaceutical ingredients (API) over the gastrointestinal track (GIT). American surveys revealed that 8% of patients skip doses and 4% discontinue therapy because of difficulties in swallowing tablets [6]. To overcome these issues, orodispersible films (ODFs) are considered to be an alternative drug form for tablets or capsules. ODFs dissolve or disintegrate in the mouth after contact with saliva (no water is needed for this purpose) and form a solution or suspension that may be easily swallowed. Breaking down into soft particles in the mouth prevents discomfort for patients. Moreover, the addition of flavor enhancers or the use of API flavor-masking technologies can greatly increase the preference for this form of the drug, especially in children [7,8]. The delivery of drugs via fast dissolving films is also an effective alternative for drugs with low bioavailability when administered by other routes [9,10,11,12,13,14]. Finally, ODFs are excellent systems for personalized therapy, especially for APIs with a narrow therapeutics index [15]. In these individualized medicines concepts, ODFs may be produced in hospitals or community pharmacies, tailored to the patient’s needs [16,17]. It makes sense, mostly due to many technological issues that may happen during the manufacturing of ODFs on a big scale. The most common problems in this field worth mentioning are homogeneity issues and batch-to-batch variability. Despite these limitations, the ODFs are still gaining popularity. The conception of this drug form dates back to 1955. Nevertheless, the real interest on a big scale started in 2001 with the launching of Listerine by Pfizer [18]. Since then, ODFs have been successfully developed which resulted in a wide range of products becoming available on the markets around the world. ODFs are carrier not only for typical API such as Sildenafil, Ondansetron, or Zolpidem, but also for vaccines [19], probiotics [20], herbal extracts [21], and nutrients such as vitamins [22]. Improving the bioavailability of some APIs was a trigger for implementing solutions that enable controlling the rate of drug release. In this way, ODFs became a carrier for microparticles [23], nanoparticles [24], or nanocrystals [18], and self-emulsifying systems [25]. The development is also visible in another aspects, namely in manufacturing methods. Among them, solvent casting is the most popular, but there are also other methods such as hot-melt extrusion or electrospinning and very promising printing technologies (two-dimensional ink-jet printing, three-dimensional, additive printing, and flexographic printing methods) [18]. The combination of the above-mentioned methods or their different varieties are often patented and therefore will probably determine the future of ODFs.

European Pharmacopoeia (Ph.Eur.) defines ODF as melting films of one or multiple layers formulated with suitable materials, that are put in the mouth where they rapidly dissolve [26]. ODFs have an important beneficial feature, which is the avoiding of the hepatic first pass effect. As APIs are released and mostly absorbed in a mouth, the bigger doses of APIs may enter systemic circulation, compared to GIT (where the reduction of the dose is caused by hepatic activity), and a smaller side-effect may occur due to less metabolites being produced in the liver. Nowadays, extensive research is conducted using many APIs, which may contribute to the application of ODFs in the treatment of an increasing number of diseases. New formulations are developed and some of them hit or will hit the market. Recently, however, it is observed that some of the ODF preparations are discontinued [27,28].

The aim of this manuscript was to review the recent literature describing the manufacturing of ODFs with different APIs and compare it with the array of ODF available on the commercial markets. In other words, what the dependence is between APIs being presented at ODFs scientific literature and APIs available for patients through the ODF’s sale. Another goal of this review is to highlight the limitations of ODFs and present some of the technological meanings to overcome these constraints.

## 2. Technological and Formulation Overview

There are many important features of ODFs from a quality and/or technological point of view: the appearance, film thickness, tensile strength, percent elongation, drug content, disintegration time, dissolution rate, pH of ODF surface (which should be closed to pH of the oral cavity), mucoadhesiveness, moisture content, and homogeneity [29,30,31]. ODFs must also be strong and flexible at the same time. However, Ph. Eur gives a very limited requirement for ODF manufacturing and testing. The main mandatory technological requirements enclosed in Ph. Eur. are releasing time and tensile properties. This means that ODF should “possess suitable mechanical strength to resist handling without being damaged” and show a sufficient drug release time. It must be emphasized that the release time for ODFs refers to the requirements for conventional solid dosage forms which might be regarded as not adequate [26]. In turn, according to the FDA, an ODF must be small, lightweight (up to 500 mg), and must disintegrate within 30 s [32].

**Figure 1:**
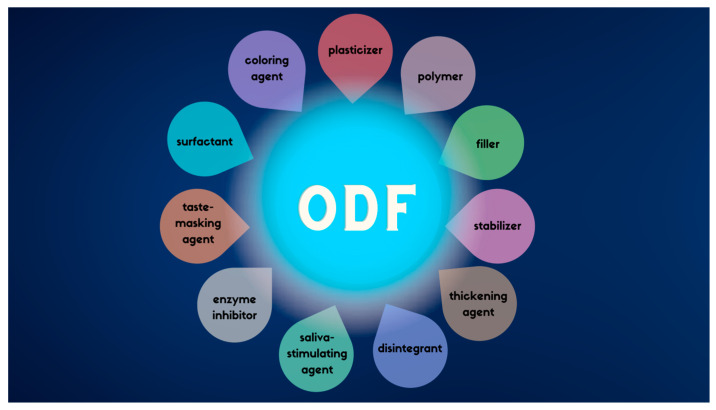
Excipients used in formulation of ODFs [33].

The ODF formulations are based on a film-forming polymers (natural or synthetic or both) and other ingredients including plasticizers (agents providing flexibility and enhanced mechanical properties), fillers, saliva-stimulating agents to increase salivation and facilitate disintegration, disintegrants or superdisintegrants, taste-masking agents to cover the bitter and unpleasant taste of many APIs, coloring agents to make the film more attractive to consumers, surfactants, enzyme inhibitors, stabilizers, and thickening agents (see Figure 1) [33]. Not all of the above-mentioned groups of substances are included in each of the manufactured ODF. Depending on the formulation (composition of excipients especially) ODF may act locally or manifest systemic action [31].

The disintegration time of ODFs is influenced by the film-forming polymer, the API particles load in the films, and other excipients [34]. As it was mentioned above, some of the excipients used in the composition of ODFs are superdisintegrants. This group most often includes (but is not limited to) the following agents: sodium starch glycolate, croscarmellose sodium, and crospovidone [35]. Their role is to decrease the disintegration time upon contact with the saliva. Unfortunately, it is challenging to obtain the disintegration time of 30 s. Zhang et al. investigated the impact of two types of superdisintegrants (sodium starch glycolate and croscarmellose sodium) on the disintegration time of ODF loaded with a poorly water-soluble drug (Fenofibrate) and observed that the disintegration time was reduced from 280 s to 160 s, which is far away from the optimal value of <30 s [35]. However, Takeuchi et al. observed that different types of insoluble additive particles (IPs), i.e., microcrystalline cellulose and partially pregelatinized starch, affected the properties of ODFs prepared with HPMC and low substituted hydroxypropyl cellulose. The addition of IPs shortened the disintegration time of ODFs to the targeted disintegration time of approximately ≤30 s, without further additives, and when a suitable number of IPs were added, the mechanical strength of ODF was not significantly weakened [36]. Similarly, Kamaran et al. investigated the impact of mesoporous silica nanoparticles (MSNs) on disintegration properties of ODFs and found that the increased number of MSNs in ODFs led to the shortening of the disintegration time of the film. Therefore, superdisintegrants may not be considered as the main factor providing the optimal disintegration time of ODFs [37,38].

The disintegration time is influenced by formulation parameters of the matrix composition (polymer, surfactants, other excipients) while dissolution is mainly based on the surface area of the embedded particles [34]. Many efforts are made to improve dissolution and bioavailability as orodispersible films are also a delivery platform for low soluble APIs. For instance, aripiprazole’s (APR) low solubility in water with its impact on the dissolution rate may be overcome by complexation with cyclodextrins [39,40] and nanosuspension formation [41]. A promising solution is also wet milling, the method that provides the reduction of API particle sizes to submicron- or nanoparticles. Because of the direct proportionality of the specific particle surface area and the dissolution rate of the API, the bioavailability may be increased. Thus, the embedding of nanoparticles in ODFs is a promising strategy to improve the bioavailability of poorly water-soluble APIs [24]. The surface area may be increased by micronisation made by ultrasonication which finally leads to greater dissolution behaviour [42]. Interesting studies were performed by Steiner et al. Five different formulation strategies were closely investigated regarding their impact on API bioavailability: the preparation of amorphous solid dispersions, embedding of the APIs in a lipid nanosuspension and a lipid nanoemulsion, and the embedding of API nanoparticles or micronized API particles in the film-forming matrix [43].

**Figure 2:**
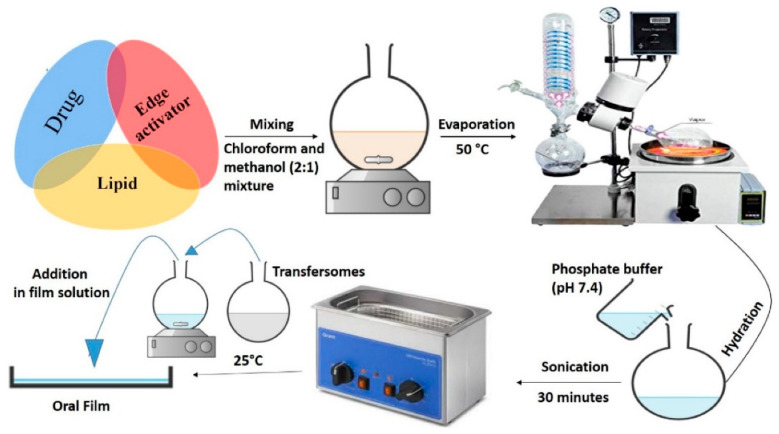
Schematic representation of the transfersomes formulation process [44].

Another interesting approach for enhancing bioavailability was proposed by Islam et al. Ebastine, which is a second BCS class drug which was incorporated into transfersomes (a specific drug carrier) in order to increase its transmucosal delivery through the gastrointestinal track. In turn, in order to improve some tranfersomes characteristics, these were integrated into oral films. This novel carrier system turned out to be effective in the delivery of poorly soluble Ebastine (Figure 2) [44].

Solid dispersions are also a common approach to increase drug dissolution. Łyszczasz et al. studied a solid dispersion of APR and Poloxamer^®^ 407 manufactured by the ball milling method. Such a dispersion was then incorporated into the orodispersible film. It was found that this action led to an over 100-fold increase in drug solubility in comparison with the pure drug. Moreover, ODFs with solid dispersion showed faster drug release (>95% below 15 min) and disintegration (<30 s), compared with films loaded with raw APR [45].

Other studies revealed that the combination of fused deposition modelling (FDM) with the preparation of the orodispersible films with poorly water-soluble substances, such as APR, led to an increased dissolution rate in comparison to casted films (prepared with solvent casting method). FDM provided full amorphization of the APR and combining with the porous structure of the printed film led to above mentioned advantage. A high concentration of PVA polymer (used for preparation of filaments) stabilized the amorphous form of APR in 3D printed films. In turn, the solvent casting process led to only partial amorphization and to the crystallization of amorphous ARP into different polymorph forms. Therefore, this API and this formulation FDM method turned out to be more advantageous than solvent casting [46]. Another example of amorphous solid dispersions (ASDs) as an effective solution to improve the oral delivery of poorly water-soluble drugs is the work of Cho et al. Three-dimensional (3D) printing based on hot-melt pneumatic extrusion (HMPE) was a method used for producing ASDs of olanzapine. Various ratios of film-forming polymers and plasticizers (polyethylene oxide, poloxamer 407, poloxamer 188, PVP VA64) were investigated to enhance the printability and optimize the printing temperature. The work conclusion was that the formulation with poloxamer 188 indicated the fastest dissolution time compared to the others, and the printed films showed immediate dissolution profiles. It was attributed to the amorphous solid dispersion of poorly water-soluble drugs and the composition of hydrophilic polymers [47].

Most of the developed ODF systems are fast-disintegrating and quick API releasing. This is a very common feature. There are only a few successful attempts of manufacturing fast disintegrating ODFs with a prolonged or modified release [48,49,50].

It must be pointed out that a typical feature of ODFs is using a single API in the whole composition. Nevertheless, in the literature one may find examples of fixed-dose combinations of two APIs. A good example of such a composed ODF is the work presented by Javed and co-workers [51]. The authors developed and optimized the production of ODF for the rapid release of sumatriptan succinate and prochlorperazine maleate. These two APIs present a clinically proven combination for treating migraines and associated nausea and vomiting [52]. Other examples of two API combinations include the study of Thummala et al. who developed ODF with ledipasvir and sofosbuvir used in the treatment of hepatitis C virus infection and the study of Thabet and co-workers who proved that it is feasible to produce ODFs containing therapeutic doses of two cardiovascular drugs (enalapril maleate and hydrochlorothiazide) in a continuous manufacturing process [9,53]. In turn, Göbel et al. proposed the modification of the solvent casting technique that aimed to divide oral films into two or more compartments that allowed for combining two or more APIs into one oral film. Successful feasibility studies for the combination of different film-forming polymers to generate the so-called tandem films were performed. It must be emphasized that the main restriction of such an approach is a relatively low API loading capacity which limits this method only for high-potent API [54].

## 3. Personalized Therapy

Oral solid doses (tablets or capsules) are often manufactured at limited dose variants on a large scale. The limited number of available doses may lead to undesired treatment results and side-effects, especially for formulations that include APIs with a narrow therapeutic index [55,56]. Therefore, in order to solve these problems, various 3D printing techniques have been applied to develop ODFs such as fused deposition modeling (FDM) [57,58], 3D inkjet printing [16], flexographic printing [18], and semi-solid extrusion (SSE) 3DP [17]. 3D printing gives the opportunity for the very precise designing of ODFs with an aid of computer software (CAD) which reduces the dose inaccuracy caused by the uneven thickness of the films during cutting [17]. 3DP was introduced into the pharmaceutical field to provide a solution for personalized drug delivery which means that patients of different ages or genders are provided with flexible and high-quality treatment. In other words, these techniques may satisfy comprehensive needs of the individual patients by a tailor-made product formulation and design [59]. Design flexibility and accurate drug loading are strong points of 3DP. Especially, SSE 3DP is a promising method applicable for the manufacturing of personalized dosage forms at room temperature without the need for the preparation of drug loaded filaments compared to other 3DP technology. That is why SSE 3DP is easier to apply to hospitals or even community pharmacies. For instance, Yan et al. successfully developed by the SSE 3DP technique individualized ODFs loaded with levocetirizine dihydrochloride in the doses of 1.25 mg, 2.5 mg, and 5 mg [17].

**Figure 3:**
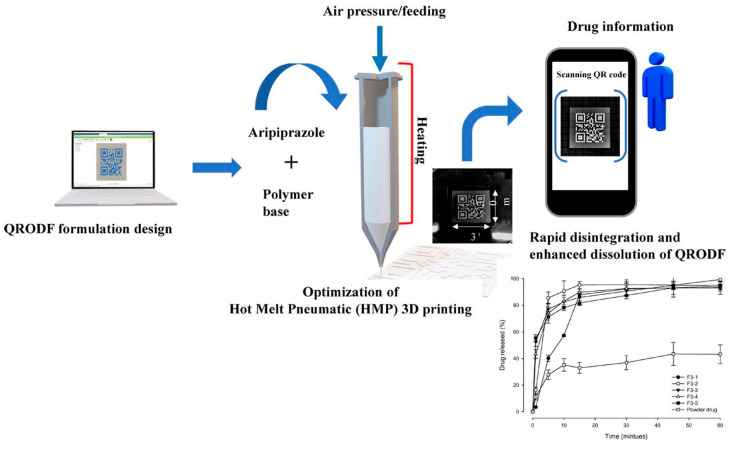
Scheme of ODF design for QR reading and rapid disintegration and enhanced dissolution [61]. This article was published in International Journal of Pharmaceutics, Vol 584, Byung-Cheol Oh, Gang Jin, Chulhun Park, Jun-Bom Park, Beom-Jin Lee, Preparation and evaluation of identifiable quick response (QR)-coded orodispersible films using 3D printer with directly feeding nozzle, 119405, Copyright Elsevier, 2020.

Steiner and co-workers proposed a very innovative approach in ODF fabrication for individualized therapy. They designed and produced drug-free ODF templates (SOFTs) that might be subsequently printed with an API solution or suspension according to the needs of the patient. The SOFTs were pervaded with pores that provided a greater API loading capacity and possessed a closed bottom side to prevent the printed fluids from passing through the film [60]. Another interesting example of using 3D printing for personalized therapy is the work of Oh et al. [61]. They prepared and evaluated a hot melt pneumatic (HMP) 3D-printed QR (Quick Response)-coded orodispersible film (QRODF) containing a poorly water-soluble APR (Figure 3). The innovative approach is that patients can easily scan the QR-encoded ODF using a smartphone and obtain information about the drug. 3D printing is also used for the preparation of tablets in the field of personalized therapy [57,62].

## 4. Limitations of ODF’s

There are many limitations of ODFs that may impede the manufacturing process [63]. As it was mentioned, one of the main disadvantages is the small drug dose that can be incorporated, due to its small size, low weight, and thin form, what in turn makes only high potent API suitable for ODF systems. However, it is known that some pharmaceutical companies had managed to develop oral films with more than 50% of drug substance per film weight (GAS-X Strips^®^) [31]. The loaded drug itself and the taste-masking agents can significantly influence the mechanical properties of the films. Therefore, the molecular weight of the film-forming material should be accurately evaluated, and specific excipients should be added to the formulation. ODFs are also not the proper drug form for APIs that are unstable at buccal pH and that may irritate the oral mucosa [7].

### 4.1. Drug Loading Capacity

Increasing the drug loading in the ODF’s with precise dose control is an issue of great need. To overcome this problem, Steiner and co-workers designed and prepared an orodispersible film template with specific pores for API by utilizing the solvent casting method and hydroxypropyl methylcellulose (HPMC) as a backbone. Consequently, the film had an outstanding drug loading capacity with a faster drug release rate [60]. The drug load of an ODF can be enhanced by increasing its size and thickness, however, this may result in thick and brittle films, with a negative influence on patient compliance [7]. Additionally, the drug load of an ODF may not be simply increased by adding a larger amount of API to the casting solution as it could negatively affect the physical properties of ODFs, such as disintegration time, thickness, and mechanical properties. Such an approach may even lead to the recrystallization of the amorphous API in the final dosage form after the evaporation of the solvent or upon storage, thereby affecting the physical properties of the product (e.g., the mechanical strength and dissolution) [64,65,66]. Takeuchi and co-workers revealed that API loading (ibuprofen, acetaminophen, or ascorbic acid) decreased the tensile strength and elastic modulus of hydroxypropylcellulose-based ODF [67].

The manufacturing process of ODFs with suspended insoluble drug substances has some serious issues, mainly due to the difficulty associated with achieving a proper homogeneity and mechanical properties of the films. Centkowska et al. found that suspended micronized loratadine particles caused dose-dependent changes in the viscosity of the casting mass and affected the mechanical quality of ODFs. However, they concluded that by using an HPMC matrix, it is possible to obtain a high load of a poorly water-soluble drug (30% of dry film mass corresponds to a dose of 5 mg per 1.5 cm^2^) with an adequate uniformity of the content but with an in vitro disintegration time below 100 s (desired value is below 30 s) [66]. Another promising strategy for high drug loading ODFs is ion-pair technology. It provides an improvement in the drug-excipient miscibility and stability in manufactured films [68,69]. The studies of Liu et al. showed that the ion-pair, especially ibuprofen-ethanolamine, improved the drug solubility in polymer up to 60% (*w*/*w*) and physical stability by 30% more than the pure ibuprofen film. Moreover, the drug dissolution rate was increased over two times. It was concluded that the high drug loading, physical stability, and dissolution rate were derived from the improved drug-polymer miscibility introduced by the formation of an ion-pair complex [70]. A further possibility for increasing the drug loading may be the application of a bi-layered (or multi-layered) ODF as a medium for an API dose dependent reduction of the viscosity of casting solutions. Visser et al. revealed the possibility of preparing a good quality bi-layered ODF using the double-casting method. The study revealed that the best disintegration time and mechanical properties were achieved by the combination of hydroxylpropyl cellulose and hypromellose, which opens perspectives for the preparation of ODFs with a higher drug load (enalapril was used as a model API in the studies) [64]. Not only the solvent casting may lead to the manufacturing of multi-layered ODFs. 3D printing is also suitable for this purpose. As an example, Elbl et al. printed multi-layered ODFs and proved that the control of the API (benzydamine hydrochloride) dose is possible by changing the thickness of the film, respectively, the overall volume of digital model, or the concentration of drug in the print dispersion [71].

### 4.2. Bitter Taste

Another critical issue is the bitter taste of many APIs since the dosage form is in direct contact with the oral mucosa. The taste masking effect of APIs may be achieved through a variety of methods and technologies, such as the simple addition of flavour and sweetener combinations [72,73] or more complex systems such as drug complexation with ion exchange resins [74] or cyclodextrins [75].

A common practice is to combine the odorous drugs and the film polymer into an inclusion compound. For instance, Liu and co-workers incorporated the donepezil hydrochloride/cyclodextrin (DP/HP-β-CD) inclusion compound into an ODF that was made of HPMC and glycerol. This approach resulted in successfully masking the peculiar smell of the drug and also provided the fast dissolving of the ODF. This study turned out to be a proper formulation strategy for the design and development of ODFs requiring taste-masking [75]. A similar approach was used by Marzouk and co-workers [76] where the complexation of fluoxetine with β-cyclodextrins led to a taste-masking effect and also by Khan et al. who focused on the developing of ODF with cefixime trihydrate and β -CD inclusion complexes [10]. Another example of manufacturing ODF with successful masking of the API bitter taste is the study of Olechno and co-workers [23,77]. An orally disintegrating film was developed with rupatadine fumarate in the form of ethylcellulose-based microparticles, using the solvent-casting method and electrospinning. Ethylcellulose is a hydrophobic polymer widely used as a taste-masking agent [78,79,80].

Multi-layered fast-dissolving oral films (MLFDFs) manufactured by 3D printing method may also pose a solution for the bitter taste of APIs. This approach assumes that a taste-masking layer is used to separate from the drug layer. After printing the drug containing layer using one of the printer heads, the taste-masking layer is printed on the drug containing layer using the other printer head [81].

It must be emphasized that the sublingual delivery of drugs has a disadvantage of a salivary washout effect. To overcome this problem, it is essential to add mucoadhesive polymers in the sublingual formulation to limit the short mucosal retention time [82]. Singh and co-authors used chitosan, an excellent mucoadhesive polymer, together with HPMC (a film forming material) to extend the mucosal retention time. Their studies indicated that the frovatriptan succinate monohydrate was successfully delivered to the sublingual mucosa, leading to a higher bioavailability [83].

### 4.3. Exposure to Humidity

It is well known that oral films are not stable in an environment with a high relative humidity [81,84]. Moreover, for many APIs, the conversion towards the hydrate forms is triggered by humidity. It is not desired, as it may cause bioavailability issues due to the variation in its solubility. Efforts are made to overcome this limitation. For example, Selmin et al. designed and manufactured ODF loaded with olanzapine using hot-melt ram extrusion printing. This method represents a reasonable alternative to the solvent casting when the polymorphic transition occurs since it limits the exposure of olanzapine to stress-factors (i.e., water and temperature) which can trigger solid-state modifications. The extruded ODFs were stable after preparation for over 30 months, which was confirmed by dissolution tests [85].

The proper water content in ODF is very crucial. Borges et al. proposed a range of 3–6% residual moisture content (RMC) for ODFs based on evaluating commercial ODFs of various compositions [29]. Foo et al. manufactured orodispersible films with an RMC of 3.4–6.2% and found that their mechanical and application properties were satisfactory [86]. The relation between RMC and disintegration time was established by Preis et al. who concluded that films of higher RMC tend to disintegrate quickly [87]. Appropriate RMC is also important for plasticizing effects, as the type and amount of plasticizers significantly impacts the flexibility which is closely related to the ease of handling and packaging [88,89]. A high amount of humidity in ODFs can also lead to the physical instability of the API that usually manifests in drug recrystallization and to increased water activity, which facilitates microbial growth [29]. The change in moisture content affects the physicochemical properties of the films. In solvent casting or the semi-solid extrusion method used for ODFs manufacturing, excipients and active ingredients are dissolved or suspended in a solution; drying is the critical step that affects the physical state of the incorporated drug and the overall properties of ODFs [88]. When improper or uncontrolled drying is used, unstable or therapeutically unsuitable polymorphs may be present in final ODFs which may reduce the treatment effectiveness or even result in adverse effects due to the presence of toxic polymorphs [90]. Janigová et al. found statistically significant impacts of drying time on thickness, moisture content, hardness, deformation at hardness, work at hardness, peak load, tensile work, and tensile strength. However, the statistical evaluation of ODFs properties showed that weight, disintegration, and elongation differences are not dependent on the drying setup [91].

### 4.4. Manufacturing Method Limitations

There are many manufacturing methods suitable for producing ODFs with more or less significant limitations. Solvent casting is the most popular [18,92,93]. The other commonly utilized methods are hot-melt extrusion, 3D printing, or electrospinning. Each of the methods pose some limitations but each of them is not dedicated for every API [63,94]. In other words, the type and features of API determine the ODF manufacturing technique. For instance, hot-melt extrusion is not applicable for thermal sensitive APIs and it is also a process of high energy consumption [95]. Similarly, FDM 3D print and electrostatic spray deposition that require high processing temperatures lead to the decomposition of sensitive actives. Electrospinning, like solvent casting, requires organic solvents which is not desired due to environmental issues [96,97]. The most popular ODF manufacturing method, which is solvent casting, as it was mentioned, may induce undesired polymorphic transitions due to the presence of humidity [85]. Moreover, the solvent casting method due to the incomplete drying process of the films induces organic solvent residue that potentially may cause the undesirable alteration of the membrane structure and changes in the drug dissolution profile [98]. Additionally, the API within the film may potentially interact with the applied organic solvent. The solvent casting method is also challenged by other issues such as trapped air bubbles, the inappropriate viscosity of casting solutions, insufficient uniformity of content, batch-to-batch variability, mingling, film shrinkage, and rippling effect [18,99]. The uniformity of films prepared by solvent casting is significantly affected by variations in the formulation of the cast solutions or suspensions. Zayed and co-workers fabricated ODFs containing domperidone based on PVP K-90 and found that different formulations of the films greatly impacted their physical and mechanical properties, flexural resistance, thickness, that further affected the drug release rate [100].

Inkjet printing and FDM 3D printing are methods that are not easily implemented in mass production. Therefore, it is currently not possible to manufacture batches on a production scale using these methods. However, 3D printing could reduce the development or manufacturing time compared to hot-melt extrusion or solvent-casting [81]. Common limitations of printing methods are nozzle clogging, smearing effect, buckling effect, and satellite effect, which are wider described in the review work of Gupta et al. [18]. Inkjet printing is suitable for temperature-sensitive APIs, however, recrystallisation issues at the nozzle plate and clogging by particles out of the processable range may appear [101]. Another drawback of the inkjet printing is a critical shortage of available printing inks that are suitable for most of the drugs and used excipients [102].

## 5. ODFs in Science and in Markets

ODFs occupy a leading place in drug technology, as they are an excellent alternative to the rapid administration of a drug substance [103]. Their origins date back to the 1970s, but the breakthrough was the launch of mouth refreshing over the counter (OTC) Listerine PocketPaks^®^ (by Pfizer). The products quickly gained the interest of patients, and ODFs were recognized as a good drug delivery platform [104,105,106,107]. The Pfizer product was a huge success, surpassing $175 million in one year [31]. Other OTC ODF films were introduced in a short time. Chloraseptic^®^ Relief Strips were introduced in 2003, which, due to the benzocaine content, provided quick relief from a sore throat. Triaminic^®^ Thin Strips (hydrobromide dextromethorphan, phenylephrine hydrochloride) and Theraflu^®^ Thin Strips (diphenhydramine hydrochloride) oral films were introduced in 2004 by Novartis for the treating of symptoms of the common cold. These Novartis preparations were recognized as the best products of 2004 [31,108]. However, these products are no longer available on the market. The initial success of the Triaminic^®^ film did not, however, help to maintain subsequent good sales. This was probably influenced by the introduction of restrictions in 2007 that declined the consumption of cough and cold medications [31,109]. Therefore, in 2012, the company decided to discontinue the distribution of Triaminic^®^ [110,111].

Apart from OTC ODFs, prescription preparations (Rx) appeared on the market later. In 2010, ondansetron ODFs, Zuplenz^®^, and Setofilm^®^ were approved both in Europe and in the USA. In a very short time, already one month after the launch of Zuplenz^®^, MonoSol together with Reckitt Benckiser Pharmaceuticals obtained FDA approval for manufacturing ODF containing buprenorphine and naloxone—Suboxone^®^ [111,112]. This product also contributed to the further development of the ODF market. Suboxone^®^ was a market success with sales exceeding $ 1.3 billion in its first year in the US [31]. The sales success has proven profitability in the Rx market and has tempted other companies to develop similar technologies, e.g., Lvogen Pine Brook, Actavis, Intelgenx, and BioDelivery Sciences International [113,114]. However, none of the preparations of the above-mentioned companies are available on the market, as a patent infringement lawsuit was filed by Reckitt Benckiser [115]. Moreover, not all of the products were accepted by patients. An example is Sudafed PETM Quick Dissolve Strips (Pfizer) containing phenylephrine hydrochloride. The sales results were probably lower on one hand due to strong competition in the cough and cold products segment, and on the other hand administration restrictions on the certain cough and cold medications introduced at that time [31].

The developing and manufacturing of new APIs is a very costly endeavor, not only in terms of funding, but it is also time-consuming. Therefore, the development of new drug delivery technologies containing the already available APIs is very beneficial and competitive [31]. Many research units around the world deliver proof that the manufacturing of ODFs with a help of different techniques and different types of polymers used as backbones is feasible (Table 1). During these studies, a large number of APIs are engaged as model drugs or targeted active substances that finally might be considered as ready-for-use patented approaches implemented in the markets around the world [18,116]. The success of Listerine PocketPaks^®^ encouraged pharmaceutical companies to invest their money into the ODF research field [31]. Consequently, many scientists developed new ODF formulations using a wide range of APIs, among which one that are worth mentioning are (but not limited to) those used in the treatment of schizophrenia, Parkinson disease, Alzheimer disease, insomnia, hypertension, migraine, allergy, diarrhea, tuberculosis, and erectile disfunctions (Table 1). As it was mentioned above, for many reasons, the type of used API determines the choice of the manufacturing method (like solvent casting, 3D printing, or hot melt extrusion) [94]. However, it is possible to design and produce ODFs loaded with a particular API using different methods. The good example is APR-loaded ODFs manufactured with solvent casting or 3D printing [46,117]. The other examples are ODFs with benzydamine hydrochloride and warfarin (solvent casting and 3D printing methods) [15,71,118].

**Figure 4:**
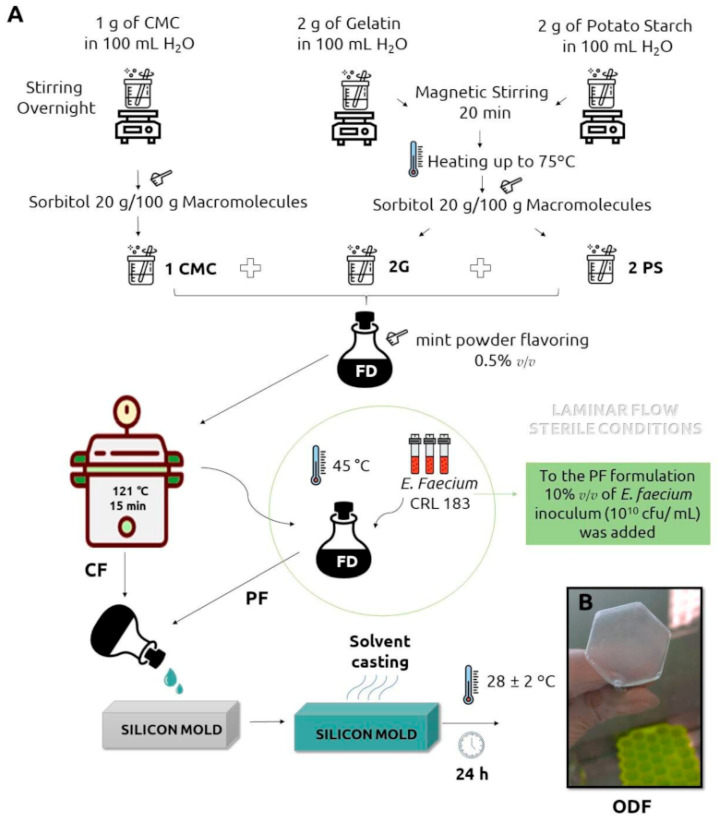
(**A**) Preparation protocol of ODF (**B**) containing Enterococcus faecium [20].

Nowadays, many advantages regarding the orodispersible forms (also called oral strips) translate into the growing interest of manufacturers. Many pharmaceutical companies launched ODF products (both prescription drug and OTC) onto the markets during the last decades. Recent estimates claim that the ODF market will grow to more than $1.3 billion per annum in the next few years [138]. Examples of ODFs commercially available on the markets around the world are summarized in Table 2. Currently, commercially available ODFs are used as a carrier of probiotic bacteria (Figure 4), proteins or vaccines, nutrients and herbal products [20,139]. The distribution of ODFs to the different countries depends on many factors and often one product sold in one country may be not available in another one. Nevertheless, a thorough review of the available literature and drug databases shows that some of the preparations are no longer available. Some of the withdrawn preparations are presented in Table 3. There are several factors that cause this situation, and the commercial ones are crucial. Most of orodispersible films are simply more expensive than other traditionally administrated forms, such as tablets or capsules, which makes the sale unprofitable [140]. Another example of a commercial factor that may cause product withdrawal is the expiry of the patent [141]. In this way, some previously commercially available products (rather expensive for patients) may disappear from the market as a consequence of the appearance of their cheaper substitutes. In other words, companies discontinue some products after the expiry of the patent, as it is no longer profitable for them. The lack of the product on the particular market may also be triggered by other non-commercial factors, especially quality aspects, side-effects, stability issues, or law restrictions.

The active substances contained in ODFs belong not only to the group of conventional drugs, but also to groups such as vaccines, probiotics, substances of plant origin, or various nutrients, as well as vitamins [18,22]. Attempts have even been made to incorporate dried plant extracts [21]. Nutraceuticals are dietary supplements used to improve health, delay aging, prevent disease and support the proper functioning of the human body. Currently, nutraceuticals are gaining a lot of interest due to their nutritional and therapeutic potential [168]. Manufacturers have recognized this market need, which is why more and more different nutraceuticals are produced in the form of ODF (Table 4). Moreover, there are companies (so called third parties) that produce and supply oral films to other pharmaceutical entities. One of these companies produces more than 15 million films a day [169].

Apart from products summarized in Table 2 and Table 4, the growing number of researches on ODFs dedicated for small scale (hospitals, pharmacies) is also worth mentioning. It seems to be a good alternative for traditional tablets that are available in a limited number of doses and mainly manufactured on a big scale and are not preferred by infants or elderly patients who require more individual doses not available on the markets [16,17]. ODFs allow for the adjustment of doses to individual needs [8,23]. In a randomized controlled trial involving the youngest children, ODFs were found to be a safe alternative to liquid drugs that could provide promising clinical results [170]. Moreover, the addition of flavor enhancers or the use of API taste masking technology may significantly increase preferences for this form of the drug, especially in children [7,8]. It is also worth noting that once the ODF formulation is placed in the mouth, it tends to stick to the mucosa before disintegrating. Thanks to this feature, patients (especially children, the elderly, or the mentally ill) cannot spit out the drug, which may happen after taking a conventional tablet [171].

**Table 4:** Examples of ODF nutraceuticals.

Drug Name	The Active Substance	Dose	Region	Producer	Drug Action/Application	References
Ashwagandha	Ashwagandha	- 100 mg	Indie/worldwide	Aavishkar	Anti-inflammatory, antioxidant	[172,173]
Astaxanthin	Astaxanthin	- 4 mg	Indie/worldwide	Aavishkar	Antioxidant	[172,173]
B 12	B 12	- 1000 mcg	Indie/worldwide	Aavishkar	Supplementation	[172,173]
B 12	Biotin, D3, Folic acid	- Biotin 5000 mcg - D3 400 IU - Folic acid 400 mcg	Indie/worldwide	Aavishkar	Supplementation	[172,173]
Breath Freshener	Spearmint, Peppermint		Indie/worldwide	Aavishkar	Refreshing the breath	[172,173]
Caffeine	Caffeine, L-Theanine, B12	- Caffeine 50 mg - L-Theanine 15 mg - B12 1000 mcg	Indie/worldwide	Aavishkar	Supplementation	[172,173]
Clove oil	Clove oil	- 8 mg	Indie/worldwide	Aavishkar	Supplementation	[172,173]
CoEnzyme Q-10	CoEnzyme Q-10	- 100 mg	Indie/worldwide	Aavishkar	Supplementation	[172,173]
Cold	AP-Bio—Andrographis Paniculata	- 100 mg	Indie/worldwide	Aavishkar	Supplementation	[172,173]
Curcumin	Curcumin	- 100 mg	Indie/worldwide	Aavishkar	Supplementation	[172,173]
Echinacea	Echinacea, D3, Manuka Honey Propolis	- Echinacea 100 mg - D3 400 IU - Manuka Honey Propolis 5 mg	Indie/worldwide	Aavishkar	Supplementation	[172,173]
Elderberry	Elderberry	- 60 mg	Indie/worldwide	Aavishkar	Supplementation	[172,173]
Expectorant	Hedera Helix	- 16 mg	Indie/worldwide	Aavishkar	Supplementation	[172,173]
D3	D3	- 400 IU	Indie/worldwide	Aavishkar	Supplementation	[172,173]
GC Well Being ODF	Sodium Selenite Pentahydrate	- 0.33 mg	South Korea	C.L.Pharm Co., Ltd.	Support deficiency that cannot be supplemented through nutrition supply	[143]
Glycine	Glycine, B1, B6, B12	- Glycine 100 mg - B1 1.6 mg - B6 2 mg - B12 100 mcg	Indie/worldwide	Aavishkar	Supplementation	[172,173]
Iron	Iron	- 14 mg	Indie/worldwide	Aavishkar	Supplementation	[172,173]
K2 + D3	K2 + D3	- K2 70 mcg - D3 1000 IU	Indie/worldwide	Aavishkar	Supplementation	[172,173]
Listerine Pocketpaks	Eucalyptol, Thymol, Menthol		USA	Johnson&Johnson	Freshening breath	[174]
Lutein	Lutein, Zeaxanthin	- Lutein 10 mg - Zeaxanthin 2 mg	Indie/worldwide	Aavishkar	sSpplementation	[172,173]
Lysozyme	Lysozyme, B6	- Lysozyme 20 mg - B6 500 mcg	Indie/worldwide	Aavishkar	Supplementation	[172,173]
Melatonin	Melatonin, Valerian, B6	- Melatonin 5 mg - Valerian 25 mg - B6 5 mg	Indie/worldwide	Aavishkar	Supplementation	[172,173]
Multivitamin	Vitamin: A, B5, B6, B7, B9, C, B12, D3, K2, Iodine	- Vitamin: - A 5000 IU - B5 10 mg - B6 2 mg - B7 300 mcg - B9 400 mcg - C 30 mg - B12 1000 mcg - D3 400 IU - K2 80 mcg - Iodine 150 mcg	Indie/worldwide	Aavishkar	Supplementation	[172,173]
Probiotics	Bacillus Coagulans	- 5 Bill CFU	Indie/worldwide	Aavishkar	Supplementation	[172,173]
Resveratrol	Resveratrol	- 100 mg	Indie/worldwide	Aavishkar	Antioxidant	[172,173]
Spice Mix	Cinnamon, Turmeric, Bee Propolis, Garlic, Piperine, Fennel, Fenugreek, Cardamon oil, Clove oil, Capsaicin, Tulsi, Saffron		Indie/worldwide	Aavishkar		[172,173]
Snoreeze Oral Strips	Peppermint oil, Tocopheryl acetate, Menthol		PL, UK, Europa	PASSION FOR LIFE HEALTHCARE	Snoring relief	[175]
Tusheel ODF	Hedera helix L., Folium	- 16 mg of dry extract of ivy leaves	EU	Heel	As an expectorant in case of a productive cough	[176]
Vitafol (Strips)	- Vitamin B6 - Folic Acid - Vitamin B12 - Vitamin D	- Vitamin B6 2.5 mg - Folic Acid 1 mg - Vitamin B12 12 mcg - Vitamin D 1000 IU	USA	Exeltis	Prenatal vitamin	[177]

## 6. Future Perspectives

The combination of the discovering of the new and continuous improvement approach is at the heart of the pharmaceutical industry. It allows us to overcome barriers and limitations, providing progress in drug product development and also hope and a better future for patients. The number of pending patent applications and granted patents [18] with their innovative approaches make us believe that the progress in the production of ODFs is unquestionable. The future of ODFs seems to have a dual nature. On the one hand, manufacturing on a big scale is necessary because it provides access to ODFs for a large number of patients. On the other hand, personalized therapy for a small scale is a promising approach for patients that need individual therapy. Both of the development ways have their limitations that need to be overcome in the future. Currently the solvent casting method is the main method used for a big scale and there are no signs that something will change in this matter. It is possible that 3D printing would be a main manufacturing method used for personalized therapy in hospitals and pharmacies. To allow that, the introduction of a regulatory pathway that permits the development and use of personalized medical products is necessary. Another thing that needs to be achieved is the overcoming of the limited loading of ODFs and providing a harmonized method for quality control. There is no doubt that the progress in the developing of ODFs will continue as there are many groups of patients with swallowing problems (elderly, pediatric, schizophrenia, and many others) for which this drug form is the most convenient. A need for tailored doses as well as dosage forms that are easy to administer is a trigger for further development.

## 7. Conclusions

Taking a closer look at the ODF products, it can be concluded that their popularity is at its beginning. Adjusting the therapeutic dose to a specific patient, the variety of APIs used, the diversity of production methods (not only on an industrial scale but also in community and hospital pharmacies) give countless possibilities for further preparations. The limitations presented above can only contribute to the improvement of the quality and durability of the manufactured preparations and suggest marketing strategies to pharmaceutical manufacturing companies. ODFs are yet an untapped potential among drug forms.

## Figures and Tables

**Table 1:** Recent ODFs publications and utilized APIs.

Disease or Therapeutic Indications or Drug Action	API	Manufacturing Method	Film-Forming Polymers	References
Painkiller	Acetaminophen	Hot-melt ram-extrusion 3D printing	Maltodextrins	[119]
Painkiller	Acetaminophen	FDM 3D printing	PVA	[81]
Antipyretic	Acetylsalicylic acid	Electrospinning	Pullulan, chitosan	[120]
Oropharyngeal candidiasis	Amphotericin B	Solvent casting	Dextrose-derived polymers; HMPC/HPC	[121]
Schizophrenia	Aripiprazole	Solvent casting	PVA	[45]
Schizophrenia	Aripiprazole	3D printing	PEO	[61]
Schizophrenia	Aripiprazole	3D printing and solvent casting	PVA	[46]
Schizophrenia	Aripiprazole	3D printing and solvent casting	PVA, HPC	[117]
Schizophrenia	Aripiprazole	3D printing and solvent casting and electrospinning	PVA	[122]
Hypertension	Atenolol	Solvent casting	HMPC/CMC-Na/Na-alginate	[123]
Antiseptic	Benzydamine hydrochloride	Solvent casting	Maltodextrin	[118]
Antiseptic	Benzydamine hydrochloride	3D printing	Maltodextrin	[71]
Hypertension	Captopril	Solvent casting	HPMC	[124]
Hypertension	Carvedilol	Electrospinning	PVPK30, hydroxypropyl-β-cyclodextrin	[125]
Infections of the respiratory tract	Cefixime trihydrate	Solvent casting	HPMC	[10]
Depression	Citalopram	Solvent casting	HPMC, Okra biopolymer	[126]
Anti-inflammatory	Diclofenac sodium	Solvent casting	HPMC E3	[72]
Anti-inflammatory	Diclofenac sodium	Solvent casting	HPMC	[50]
Angina and hypertension	Diltiazem hydrochloride	Solvent casting	HPMC, CMC	[127]
Alzheimer’s disease	Donepezil hydrochloride	Solvent casting	HPMC, hydroxypropyl-β-cyclodextrin	[75]
Alzheimer’s disease	Donepezil hydrochloride	Solvent casting	HPMC	[36,74]
Allergy	Ebastine	Solvent casting	HPMC	[44]
Migraine	Eletriptan hydrobromide	Solvent casting	PVA	[11]
Hypertension	Enalapril	Solvent casting	HPMC, HPC, HEC	[64]
Hypertension	Enalapril and hydrochlorothiazide	Solvent casting	HPC/PVA	[53]
Migraine	Frovatriptan succinate monohydrate	Solvent casting	HPMC E15, chitosan	[83]
Painkiller, anti-inflammatory	Ibuprofen	Solvent casting	HPC	[67]
Painkiller, anti-inflammatory	Ibuprofen	Solvent casting	PVP	[70]
Tuberculosis	Isoniazid	Electrospinning	Pullulan/HPMC	[128]
Painkiller	Ketoprofen	Solvent casting	HPMC	[129]
Hepatitis C virus infection	Ledipasvir and sofosbuvir	Solvent casting	MC (methyl cellulose)	[9]
Allergy	Loratidine	Solvent casting	PVA/PVP; HMPC	[66]
Heart diseases	Metoprolol	Inkjet printing	HPMC	[101]
Depression	Mirtazapine	3D printing	HPMC	[130]
Painkiller, anti-inflammatory	Naproxen, anthraquinone	Solvent casting	Pharmacoat 606 (HPMC)	[34]
Schizophrenia	Olanzapine	Hot-melt ram-extrusion 3D printing and solvent casting	Maltodextrin	[85]
Schizophrenia	Olanzapine	3D printing based on hot-melt pneumatic extrusion (HMPE)	Polyethylene oxide, poloxamer 407, poloxamer188, PVP VA64	[47]
Therapeutic proteins	Ovalbumin, lysozyme, β-galactosidase	Solvent casting	Pullulan, trehalose	[131]
Adrenal insufficiency	Prednisolone	Solvent casting	PVA	[37]
Adrenal insufficiency	Prednisolone sodium phosphate	Electrospinning	PVA	[132]
Candida spp. infections	Probiotic bacteria Enterococcus faecium CRL183	Solvent casting	CMC	[20]
Diarrhea	Racecadotril	Solvent casting	PVA	[133]
Anti-migraine	Rizatriptan	Solvent casting	Maltodextrin, pullulan	[134]
Parkinson’s disease	Ropinirole HCl	Solvent casting	HPMC 603	[14]
Allergy	Rupatadine fumarate	Solvent casting	HPMC	[23]
Allergy	Rupatadine fumarate	Electrospinning	HPMC	[77]
Migraine and associated nausea and vomiting	Sumatriptan-prochlorperazine	Solvent casting	PA	[51]
Muscle realaxant	Tizanidine hydrochloride	Solvent casting	Chitosan-alginate	[135]
Erectile dysfunction	Vardenafil	Solvent casting	PVP/MC/sodium alginate (SA)/polyvinylpyrrolidone K 30 (PVP)	[73]
Anticoagulant	Warfarin	Solvent casting (continuously working pilot-scale coating bench)	HPMC, HPC, PVA	[136]
Anticoagulant	Warfarin	3D printing	PVA, HPC	[15]
Anticoagulant	Warfarin	Semi-solid extrusion 3D printing and 2D inkjet printing	HPMC	[16]
Insomnia	Zaleplon	Solvent casting	Lycoat^®^ RS 720	[137]

**Table 2:** Examples of prescription and OTC ODF drugs.

Drug Name	The Active Substance	Dose	Region	Producer	Drug Action/Application	References
Amlodipine OD Film “QQ”	Amlodipine Besilate	- 2.5 mg - 5 mg	Japan	Kyukyu Pharmaceutical Co., Ltd.	Hypertension, angina pectoris	[142]
Cendom ODF	Tadalafil	- 10 mg - 20 mg	South Korea	C.L.Pharm Co., Ltd.	Treatment for erectile dysfunction	[143]
Chloraseptic strips	Benzocaine, menthol	- 3 mg	USA	Prestige Brands	Sore throat relief	[144]
Cool Strip ODF	Cetylpyridinium Chloride	- 1.5 mg	South Korea	C.L.Pharm Co., Ltd.	Pharyngitis, tonsillitis, and stomatitis treatment	[143]
Donepezil Hydrochloride OD Film “EE”	Donepezil Hydrochloride	- 3 mg - 5 mg - 10 mg	Japan	Kyukyu Pharmaceutical Co., Ltd.	Inhibiting the progression of dementia symptoms associated with dementia of the Alzheimer’s type and dementia with Lewy bodies	[142]
Fluor-I-Strips A.T.	Fluorescein Sodium	- 9 mg	USA	Wyeth Ayerst Laboratories	Specially prepared sterile ophthalmic strip for diagnostic use	[145]
Hemoramin ODF	Folic acid, Ferric Hydroxide Polymaltose	- Folic acid 150 μg - Ferric Hydroxide Polymaltose 50 mg	South Korea	C.L.Pharm Co., Ltd.	Prevention and treatment for iron-deficiency anemia	[143]
Ignis ODF	Sildenafil	- 50 mg	South Korea	C.L.Pharm Co., Ltd.	Treatment for erectile dysfunction	[143]
Kynmobi	Apomorphine HCl	Sublingual film 10 mg, 15 mg, 20 mg, 25 mg, 30 mg		Sunovion Pharmaceuticals Inc.	Acute, intermittent treatment of “off” episodes in patients with Parkinson’s disease	[146]
Loratadine OD Film 10 mg “MOCHIDA”	Loratadine	- 10 mg	Japan	Kyukyu Pharmaceutical Co., Ltd.	Allergic rhinitis, urticaria, itch associated with skin diseases (eczema/dermatitis, pruritus cutaneous)	[142]
Neutoin ODF	Donepezil HCl	- 5 mg - 10 mg	South Korea	C.L.Pharm Co., Ltd.	Treatment of dementia	[143]
New Pezil ODF	Donepezil	- 5 mg - 10 mg	Korea	C.L.Pharm Co., Ltd.	Treatment for Alzheimer—dementia	[143]
Olopatadine Hydrochloride OD Film “MARUHO”	Olopatadine Hydrochloride	- 2.5 mg - 5 mg	Japan	Kyukyu Pharmaceutical Co., Ltd.	Allergic rhinitis, urticaria, itch associated with skin diseases	[142]
Ondansetron Rapidfilm ODF	Ondansetron	- 4 mg - 8 mg	Germany	Applied Pharma Research/Labtec	Prevention and treatment of chemo- and radiotherapy induced nausea and vomiting	[147]
Ora-film	Benzocaine Strips		USA		Treats mouth sores.	[148]
Rizaport	Rizatriptan Film	- 10 mg	Spain	Intelgenx	Selective 5-HT1B/1D receptor agonist indicated for the treatment of migraines	[149]
Sentrip ODF—tadalafil	Tadalafil	- 10 mg - 20 mg	Korea	C.L.Pharm Co., Ltd.	Treatment for erectile dysfunction	[143]
Setofilm ODF	Ondansetron	- 4 mg - 8 mg	Europe, Australia, and New Zealand	Norgine	Prophylaxis and treatment of acute and delayed nausea and vomiting	[150]
Suboxone	Buprenorphine and Naloxone sublingual film	Buprenorphine/Naloxone: - 2 mg/0.5 mg - 4 mg/1 mg - 8 mg/2 mg - 12 mg/3 mg	USA, UE	Indivior UK Limited	Treatment of opioid dependence	[151]
Sympazan	Clobazam oral film	- 5 mg - 10 mg - 20 mg		Aquestive Therapeutics	Treats the symptoms of seizures	[152,153]
Vinix ODF	Sildenafil	- 50 mg - 100 mg	Korea	C.L.Pharm Co., Ltd.	Treatment for erectile dysfunction	[143]
Voglibose OD Film “QQ”	Voglibose	- 0.2 mg - 0.3 mg	Japan	Kyukyu Pharmaceutical Co., Ltd.	Improvement of postprandial hyperglycemia in diabetes mellitus	[142]
Zentrip	Meclizine hydrochloride	- 25 mg		Sato Pharmaceutical Co., Ltd.	Prevention and treatment of the nausea, vomiting, or dizziness associated with motion sickness	[154]
Zolpidem Tartrate OD Film “MOCHIDA”	Zolpidem Tartrate	- 5 mg - 10 mg	Japan	Kyukyu Pharmaceutical Co., Ltd.	Insomnia	[142]
Zuplenz	Ondansetron film	- 4 mg - 8 mg		Aquestive Therapeutics	Prevention of nausea and vomiting associated with highly emetogenic cancer chemotherapy, including cisplatin ≥ 50 mg/m^2^	[155]
Zyris ODF	Tadalafil	- 20 mg	South Korea	C.L.Pharm Co., Ltd.	Treatment for erectile dysfunction	[143]

**Table 3:** Discontinued ODFs.

Drug Name	The Active Substance	Dose	Region	Producer	Drug Action/Application	References
Antimal ODF	Primaquine	- 15.0 mg	Korea	C.L.Pharm Co., Ltd.	Antiprotozoal	[156]
Benadryl Allergy Quick dissolve strips	Diphenhydramine HCl	- 25 mg	USA, EU	McNeil Consumer Healthcare Division of McNeil-PPC, Inc	Temporarily relieves these symptoms due to hay fever or other upper respiratory allergies	[157]
Donepezil HCl Hexal SF	Donepezil	- 5 mg - 10 mg	EU	Hexal AG	Treatment of mild to moderately severe Alzheimer’s dementia	[158]
Gas-X thin strips	Simethicone	- 62.5 mg		Novartis Consumer Health	Flatulence	[159]
Hiforce 100 ODS	Sildenafil	- 100 mg	Indie	Healing Pharma	Treatment for erectile dysfunction	[160]
IvyFilm	Dried extract from Ivy leaves (*Hedera helix* L., folium);	- 16 mg	Africa	LAMAR INTERNATIONAL	Loosens mucus in the airway	[161]
Jack & Jill Thin Strips Cough	Dextromethorphan	- 7.5 mg	USA, Canada	The Buckley’s Company	Antitussives	[162]
Niquitin strips	Nicotine	- 2.5 mg	EU	Omega Pharma Ltd.	Relieve and/or prevent craving and nicotine withdrawal sympoms associated with tobacco dependence	[163]
Olanzapin Hexal SF	Olanzapine	- 10 mg - 15 mg	EU	Hexal AG	Schizophrenia	[158]
Orajel Kids Sore Throat Relief Strips	Pectin	- 28 mg	Canada, USA	Church & Dwight, Inc.	Soothes pain & irritation of throat	[164]
Pedia Lax Quick dissolve strips	Standardized sennosides	- 8.6 mg	USA	Fleet Company	Constipation	[165]
Ramea ODF	Ramosetron HCl	- 0.1 mg	Korea	C.L.Pharm Co., Ltd.	Antiemetic	[156]
Risperidon Hexal SF	Risperidone	- 0.5 mg - 1 mg	EU	Hexal AG	Schizophrenia	[158]
Sudafed PE Quick dissolve strips	Phenylephrine HCl		USA	Pfizer	Decongestant	[166]
Theraflu Thin Strips multi symptom	Diphenhydramine hydrochloride	- 25 mg;	USA	Novartis Consumer Health	Treat symptoms of allergies and the common cold.	[28]
Triaminic Thin Strip	Dextromethorphan HBr/Phenylephrine HCl	- Dextromethorphan HBr 5 mg - Phenylephrine HCl 2.5 mg	USA	Novartis Consumer Health	Cough suppressant, nasal decongestant	[27]
Zolmitriptan Renantos Schmelzfilm	Zolmitriptan	- 2.5 mg - 5 mg	EU	Renantos	Antimigraine	[167]

## Data Availability

Not applicable.
